# Study on the Freezing Protection Effect of Melatonin on *Lactobacillus plantarum* FQR

**DOI:** 10.3390/foods15111836

**Published:** 2026-05-22

**Authors:** Yuting Feng, Yating Wu, Menglu Wang, Rui Wang, Leying Song, Lin Mei

**Affiliations:** 1 School of Food and Nutrition, Anhui Agricultural University, Hefei 230036, China; fengyuting@stu.ahau.edu.cn (Y.F.); 15055081845@163.com (Y.W.); 17681136772@163.com (M.W.); 15656095081@163.com (R.W.); 18894586954@163.com (L.S.); 2 Key Laboratory of Agricultural Product Fine Processing and Resource Utilization, Ministry of Agriculture and Rural Affairs, Hefei 230036, China

**Keywords:** melatonin, lyophilisation protectant, *Lactobacillus plantarum*, metabolism, transcriptomics

## Abstract

This study aimed to investigate the regulatory effect and cryoprotective mechanism of melatonin (MT) on the physiological functions of *Lactobacillus plantarum* FQR during freezing and freeze-drying. Results indicated that the addition of 5 mg/mL MT as a cryoprotectant maximized the freeze-drying survival rate to 32.04 ± 2.14%. MT effectively alleviated low-temperature and freeze-drying stress by reducing extracellular alkaline phosphatase activity, enhancing intracellular lactate dehydrogenase activity, and decreasing extracellular β-galactosidase activity without significant differences. Higher survival rates in defining medium further suggested that MT reduced damage to cell wall and membrane structures during lyophilisation, decreased membrane permeability, and preserved cellular physiological functions. In addition, MT supported cellular energy metabolism and protein synthesis, enhanced transmembrane potential to facilitate ATP transport, and helped maintain intracellular and extracellular pH balance. The prepared freeze-drying protectant containing 69.80 mg/mL exopolysaccharides (EPS) and 4.25 mg/mL MT showed better protective effects than the control group. MT also increased bound water content, lowered the freezing point of the solution, and inhibited ice crystal formation. Transcriptomic analysis revealed that amino acid biosynthesis, amino acid metabolism, and ABC transport systems were the primary pathways affected by MT treatment. These findings demonstrate that MT improves freeze-drying tolerance by maintaining membrane integrity, regulating cellular metabolism, and enhancing oxidative stress resistance. Given its natural biosynthetic origin, generally recognized as safe (GRAS) status, and absence of residual solvents or allergenic proteins, MT can be safely considered for incorporation into food and nutraceutical products. This study underscores the practical relevance of MT as a functional component in compound cryoprotectants, providing a feasible strategy to enhance the viability, stability, and industrial applicability of *Lactobacillus plantarum* during freeze-drying and storage.

## 1. Introduction

Melatonin (MT), also known as N-acetyl-5-methoxytryptamine, is a derivative of tryptophan and serves as a crucial endogenous hormonal signaling molecule. It regulates the growth, development, and physiological biochemical metabolism of both animal and plant cells [1], and exhibits biological activities such as antioxidant, anti-inflammatory, and anti-apoptotic effects. As a potent antioxidant, MT significantly inhibits peroxidation reactions in biomolecules, safeguards cellular structural integrity, prevents oxidative DNA damage, and reduces peroxide accumulation. It also antagonizes peroxidation reactions triggered by exogenous factors and free radical-induced cellular damage. Its cryoprotective functions encompass several aspects. In plants, MT preserves membrane integrity, modulates water status and photosynthetic performance, maintains redox homeostasis, accumulates osmoprotectants, and enhances antioxidant capacity, thereby increasing cold stress tolerance. In animals, it scavenges accumulated ROS, alleviates oxidative stress during germ cell cryopreservation, and improves post-thaw quality. In microorganisms, MT directly eliminates ROS, reduces lipid peroxidation, increases the proportion of unsaturated fatty acids, and enhances stress resistance [2]. Furthermore, MT can interact with phospholipid bilayers and maintain membrane structural stability under stress conditions, potentially improving bacterial survival [3]. Cui et al. [4] reported that adding MT combined with Tween 80 and β-carotene improved freeze-drying protection and maintained the viability of *Lactobacillus plantarum*. Despite these findings, research on MT’s protective effects during LAB freeze-drying remains limited.

Lactic acid bacteria (LAB) are Gram-positive, catalase-negative microorganisms that produce lactic acid as the primary end product of carbohydrate metabolism and are widely employed in fermented foods to enhance flavor, texture, and nutritional value. Freeze-drying is commonly used to preserve LAB, but freezing stress, ice crystal formation, membrane rupture, and dehydration during lyophilisation can compromise cell viability and function [5]. Various lyophilisation protectants, such as trehalose [6], skimmed milk powder, polysaccharides [7], amino acids, and sugar alcohols, have been applied to improve freeze-drying survival. Among these, trehalose and skimmed milk are commonly used because of their stabilizing effects on membranes and mitigation of dehydration stress. Nevertheless, conventional protectants cannot fully prevent cellular damage caused by recrystallisation and oxidative stress, highlighting the need to screen more effective cryoprotectants [8].

Against this research backdrop, this study used *Lactobacillus plantarum* FQR to investigate the protective effects and regulatory mechanisms of MT during freeze-drying. The effects of MT on cell survival, membrane integrity, physiological characteristics, and transcriptomic responses were evaluated to clarify its cryoprotective mechanism and assess its potential as a novel natural lyoprotectant for LAB preservation.

## 2. Materials and Methods

### 2.1. Materials

*Lactobacillus plantarum* FQR(CCTCC, WuHan, China) was provided by the Livestock Product Processing Laboratory and is patented and deposited at the China Type Culture Collection Centre (CCTC), with deposit number CCTCC NO: M 2021228.

### 2.2. Sample Preparation

Inoculate activated *Lactobacillus plantarum* FQR at a 2% (*v*/*v*) inoculum rate into MRS broth. Ferment at 37 °C for approximately 24 h. Centrifuge the fermented broth at 4 °C and 7000 rpm for 10 min to remove the supernatant. Wash the bacterial pellet 2–3 times with 0.9% sterile saline solution. Centrifuge the collected bacterial pellet and set aside for later use. MT solutions at concentrations of 2, 3, 4, 5, and 6 mg/mL were prepared. The MT solutions were mixed with centrifuged bacterial slurry at a 1: 1 volume-to-mass ratio. The prepared bacterial suspensions were frozen overnight at −20 °C and then freeze-dried in a vacuum freeze-dryer for 48 h.

The abstract of the article mentions that adding 5 mg/mL MT as a cryoprotectant can maximize the freeze-drying survival rate to 32.04 ± 2.14%. The freeze-drying protectant prepared with 69.80 mg/mL extracellular polysaccharide (EPS) and 4.25 mg/mL MT has a better effect than the control group. Based on the pre-experiment exploration, the MT concentration was finally determined to be 2, 3, 4, 5, and 6 mg/mL. According to the research of Passot, S. [5], Liu Zhou [6], Xue Wang [9], etc., this article decides to adopt the concentration gradient method (setting five levels of 2, 3, 4, 5, and 6 mg/mL) for the experimental design of the protectant screening. Melatonin, as a bioactive molecule, shows a significant concentration-dependent effect on microorganisms. This is an important reason why this article needs to screen the concentration range of 2–6 mg/mL. At an appropriate concentration, melatonin shows a positive protective effect on microorganisms. When the concentration is too high, melatonin may inhibit bacteria. Considering the cost factor comprehensively, the final concentration of melatonin is determined to be 2–6 mg/mL.

### 2.3. Vacuum Freeze-Drying Conditions

*Lactobacillus plantarum* FQR underwent vacuum freeze-drying experiments using a vacuum freeze-dryer. The freeze-drying process maintained a vacuum level of 0.001 mbar, with a main drying temperature of −58.8 °C and a main drying duration of 48 h.

### 2.4. Survival Rate of Freeze-Dried Lactobacillus plantarum FQR

Live bacterial counts were calculated using the plate count method. After freeze-drying, the lyophilized bacterial powder was resuspended in 0.9% sterile saline to its original volume. Bacterial suspensions were taken before and after freeze-drying, then serially diluted at 10-fold gradients. Two to three suitable dilutions were spread onto MRS agar plates and incubated at 37 °C for 48 h. Colony counts were recorded. The survival rate of freeze-dried powder is calculated using the following formula:

Freeze-drying survival rate (%) = (N_1_/N_0_) × 100 (1)

where N_0_ is the colony count before freeze-drying, and N_1_ is the colony count after freeze-drying.

### 2.5. Determination of Key Enzyme Activity in Cells After Freeze-Drying

#### 2.5.1. Preparation of Cell-Free Extracts

Methods were adapted from Wang [9] with minor modifications. Resuspend lyophilized bacterial powder in 0.9% sterile saline to original volume, then dilute to approximately 5 × 10^6^ CFU/mL. Centrifuge at 4 °C and 7000 rpm for 10 min; use supernatant for extracellular alkaline phosphatase and extracellular β-galactosidase assays. The pellet was washed twice with 0.9% sterile saline and used for intracellular lactate dehydrogenase and intracellular Na^+^K^+^-ATPase assays.

#### 2.5.2. Assay of Alkaline Phosphatase Activity

Alkaline phosphatase (AKP/ALP) was measured using the Alkaline Phosphatase Activity Assay Kit (Suzhou Keming Biotechnology Co., Ltd., Suzhou, China), following the protocol specified in the manual.

Enzyme Activity Definition: One enzyme activity unit is defined as the catalytic production of 1 μmol of phenol per minute at 37 °C by 10,000 bacteria or cells.

#### 2.5.3. β-Galactosidase Activity Assay

β-Galactosidase (β-GAL) was measured using the β-Galactosidase Activity Assay Kit from Solabio, following the protocol specified in the manual.

Enzyme activity definition: One enzyme activity unit is defined as the production of 1 nmoL p-nitrophenol per 10,000 bacteria or cells per hour.

#### 2.5.4. Lactate Dehydrogenase Activity Determination

Lactate dehydrogenase (L-LDH) was measured using the Solabio Lactate Dehydrogenase Activity Assay Kit, following the protocol specified in the manual.

Enzyme activity definition: One enzyme activity unit is defined as the catalytic production of 1 nmol of pyruvate per minute per 10,000 bacteria or cells.

#### 2.5.5. Determination of ATPase Activity

Na^+^K^+^-ATPase was measured using the Na^+^K^+^-ATPase Activity Assay Kit from Suzhou Keming Biotechnology Co., Ltd., following the specific experimental methods outlined in the instruction manual.

Enzyme activity definition: One enzyme activity unit is defined as the amount of inorganic phosphate (1 μmol) produced per hour per 10,000 bacteria or cells by Na^+^K^+^-ATPase through ATP hydrolysis.

### 2.6. Determination of the Degree of Strain Damage

Cell wall integrity, cell membrane permeability, and protein synthesis protection were evaluated according to the method of Gong et al. [10] Add high-concentration lysozyme or chloramphenicol solution, sterilized by filtration, to MRS agar at 55 °C to achieve a final concentration of 5 μg/mL as the lysozyme or chloramphenicol limiting medium concentration. Use a final concentration of 0.5 M NaCl as the NaCl limiting medium concentration, with standard MRS solid medium as the control. Live bacteria counts were performed on lyophilized samples.

Lyophilized bacterial powder was resuspended in 0.9% sterile saline to its original volume. A 100 μL gradient dilution of the bacterial suspension was evenly spread onto four MRS agar plates. After incubation at 37 °C for 48 h, colony counts were performed on each plate using the following formula:

Relative viable cell proportion (%) = (N_1_/N_0_) × 100 (2)

where N_0_ is the colony count on standard MRS solid medium; N_1_ is the colony count on lysozyme, NaCl, and chloramphenicol-supplemented medium.

### 2.7. Measurement of Metabolomics

Raw mass spectrometry files were converted to mzXML format using MSConvert tool (Proteowizard, v3.0.8789, ProteoWizard Software Foundation, Palo Alto, CA, USA). Peak detection, filtering, and alignment were performed using the XCMS software package (v3.18.0, Bioconductor, Seattle, WA, USA)to generate a quantitative list of compounds. Parameters were set as follows: bw = 2, ppm = 15, peakwidth = C (5, 30), mzwid = 0.015, mzdiff = 0.01, method = “centWave”. Compound identification was performed using public databases (HMDB, massbank, LipidMaps, mzCloud, KEGG) and an in-house compound library, with parameters set to ppm < 30 ppm. Calculate the metabolite-to-isotope internal standard ratio across different concentration gradients in mixed QC samples under both positive and negative ion modes. Construct linear curves between metabolite ratios and concentrations. Optimize the linear equation to compute relative quantitative results for metabolites in sample solutions, enabling metabolomics data analysis.

### 2.8. Determination of Transcriptomics

#### 2.8.1. RNA Extraction and Detection

The Trizol Reagent kit was employed. Total RNA quality assessment: Concentration and purity were measured using NanoDrop 2000 (Thermo Fisher Scientific, Wilmington, DE, USA), while integrity was evaluated via RNA-specific agarose gel electrophoresis or 2100 analysis Agilent 2100 Bioanalyzer and RNA 6000 Nano Kit (5067-1511, Agilent Technologies, Santa Clara, CA, USA). RNA extraction was performed.

#### 2.8.2. Library Construction and Machine Sequencing

The Zymo-Seq RiboFree Total RNA Library Kit (Zymo Research, Irvine, CA, USA) was used to remove rRNA from total RNA. Subsequently, RNA was randomly fragmented using divalent cations via ion-mediated cleavage. Using RNA as a template and random oligonucleotides as primers, the first strand of cDNA is synthesized. Subsequently, the RNA strand is degraded using RNase H, and the second strand of cDNA is synthesized in a DNA polymerase I system using dNTPs with dUTP substituted for dTTP. The double-stranded cDNA is purified, followed by double-end repair, 3′-end A-adding, and sequencing adapter ligation. USER enzyme (New England Biolabs, Ipswich, MA, USA) is then added to degrade the uracil-containing second strand of the cDNA. cDNA fragments of approximately 400–500 bp were selected using AMPure XP beads (Beckman Coulter, Brea, CA, USA), amplified by PCR, and purified again with AMPure XP beads to obtain the final library. Library quality was assessed using an Agilent 2100 Bioanalyzer (Agilent Technologies, Santa Clara, CA, USA) and the Agilent High Sensitivity DNA Kit (Agilent, 5067–4626). Total library concentration was measured using PicoGreen (Quantifluor-ST fluorometer, Promega, Madison, WI, USA, E6090; Quant-iT PicoGreen dsDNA Assay Kit, Invitrogen, Carlsbad, CA, USA, P7589). Effective library concentration was quantified via StepOnePlus Real-Time PCR System (Thermo Fisher Scientific, Wilmington, DE, USA). Multiplexed DNA libraries were normalized and pooled in equal volumes. The pooled libraries were sequentially diluted and quantified before undergoing PE150 sequencing on an Illumina sequencer for transcriptome data analysis. Differentially expressed genes (DEGs) were identified using the criteria of |log2FoldChange| ≥ 1 and false discovery rate (FDR) < 0.05. GO and KEGG enrichment analyses were performed based on significantly differentially expressed genes, and pathways with *p* < 0.05 were considered significantly enriched.

### 2.9. Data Statistics and Analysis

Statistical analysis was performed using IBM SPSS Statistics 26.0 (IBM, Chicago, IL, USA). One-way analysis of variance (ANOVA) was employed to detect significant differences between data sets. Results are presented as Mean ± SD, with *p* < 0.05 indicating statistically significant differences. Graphs and charts were generated using Origin 2018 and Excel software. Box–Behnken response surface trial data were analyzed using Design-Expert 11.

## 3. Results

### 3.1. Antifreeze Protective Effect of MT on Lactobacillus plantarum FQR

#### 3.1.1. Effect of MT on the Survival Rate of Freeze-Dried *Lactobacillus plantarum* FQR

**Figure 1:**
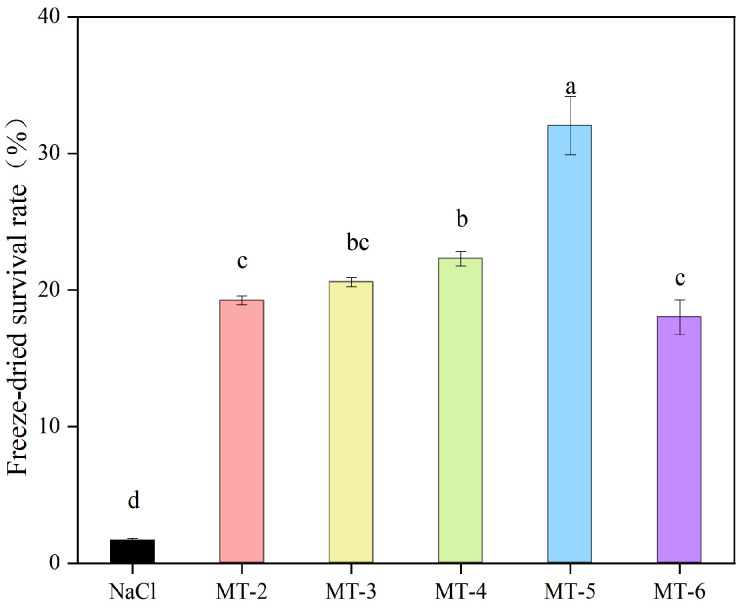
Effect of MT on the freeze-dried survival rate of *Lactobacillus plantarum* FQR. NaCl represents the lyophilization group with 0.9% sterile saline as the lyophilization protectant; MT-2, MT-3, MT-4, MT-5, and MT-6, respectively, represent the lyophilization groups with 2 mg/mL, 3 mg/mL, 4 mg/mL, 5 mg/mL, and 6 mg/mL MT as the lyophilization protectant. Different lowercase letters indicate significant differences (*p* < 0.05).

This study evaluated the cryoprotective effect of MT at concentrations ranging from 2 to 6 mg/mL on the freeze-drying survival rate of FQR (Figure 1). Compared with the NaCl group (1.70 ± 0.13%), the addition of MT significantly increased the freeze-drying survival rate of *Lactobacillus plantarum* FQR (*p* < 0.05). The survival rate showed a trend of first increasing and then decreasing with the increase in EPS concentration. Among them, the freeze-drying survival rate was the highest when 5 mg/mL MT was added (32.04 ± 2.14%).

Stefanello et al. [11] studied the freeze-drying protection effect of *Lactobacillus plantarum* fermentation on the control solution (S1 = 0.1% peptone water) and four cryoprotectant solutions: S2 (10% sucrose), S3 (5% trehalose), S4 (10% skim milk powder), and S5 (10% skim milk powder + 5% sodium glutamate). The results showed that S5 was the best cryoprotectant, with a cell survival rate of 87%, followed by S4 (74%). Luchetti et al. [12] pointed out that the cell protection mechanism of MT was related to its multifunctional properties as an antioxidant, anti-apoptotic agent, anti-inflammatory agent, and autophagy regulator. MT is considered an effective inhibitor of cellular oxidative stress because its activity as a direct antioxidant and free radical scavenger depends on its hydrogen atom transfer characteristics [13] Although there are few studies on the specific mechanism of MT on the freeze-drying protection of *Lactobacillus plantarum* FQR at present, it is speculated that MT may form a protective film in the low-temperature freeze-drying environment to reduce the damage of ice crystals to cells, and its concentration needs to be controlled within an appropriate range to achieve the best protective effect. This is consistent with the result of this study that the survival rate was the highest when the MT concentration was 5 mg/mL.

#### 3.1.2. Determination of Key Enzyme Activity in Cells After Freeze-Drying

**Figure 2:**
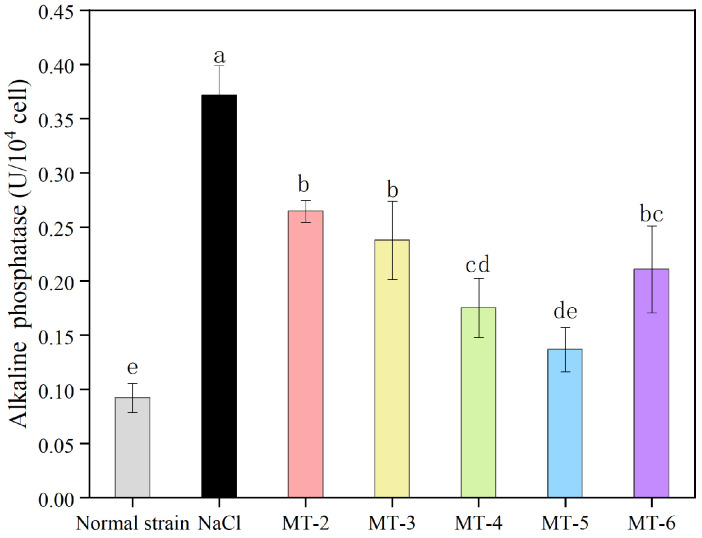
Effect of MT on extracellular AKP/ALP content of *Lactobacillus plantarum* FQR. The normal strain represents cells without lyophilization; NaCl represents the lyophilization group with 0.9% sterile saline as the lyophilization protectant; MT-2, MT-3, MT-4, MT-5, and MT-6, respectively, represent the lyophilization groups with 2 mg/mL, 3 mg/mL, 4 mg/mL, 5 mg/mL, and 6 mg/mL MT as the lyophilization protectant. Different lowercase letters indicate significant differences (*p* < 0.05).

The higher the extracellular AKP/ALP enzyme activity, the greater the cell wall damage [14]. As shown in Figure 2, the normal strain group (0.0921 ± 0.0136 U/10^4^ cell) had the lowest extracellular AKP/ALP activity before freeze-drying. The AKP/ALP concentration in the supernatant of the reconstituted freeze-dried powder increased, indicating that the cell walls of *Lactobacillus plantarum* FQR were damaged to varying degrees during freeze-drying, leading to the release of AKP/ALP outside the cell membrane. Compared with the NaCl group (0.3713 ± 0.0273 U/10^4^ cell), the addition of different concentrations of MT significantly reduced the extracellular AKP/ALP activity (*p* < 0.05). The MT concentration had a significant effect on the AKP/ALP activity of the strains after freeze-drying, showing a trend of first decreasing and then increasing with the increase in MT concentration. Among them, 5 mg/mL of MT (0.1337 ± 0.0089 U/10^4^ cell) had the best protective effect on AKP/ALP activity. After freeze-drying treatment, the extracellular AKP/ALP enzyme content in the MT group was lower than that in the NaCl group, indicating a lower level of cell wall damage. The experimental results show that the addition of MT can reduce the damage to the cell wall of the strain during freeze-drying and improve the survival ability of the strain.

**Figure 3:**
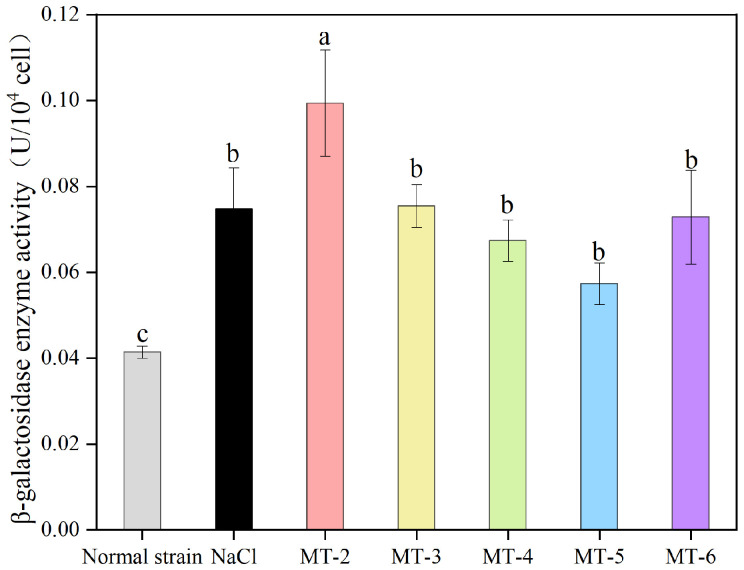
Effect of MT on extracellular β-Galactosidase content of *Lactobacillus plantarum* FQR. The normal strain represents cells without lyophilization; NaCl represents the lyophilization group with 0.9% sterile saline as the lyophilization protectant; MT-2, MT-3, MT-4, MT-5, and MT-6, respectively, represent the lyophilization groups with 2 mg/mL, 3 mg/mL, 4 mg/mL, 5 mg/mL, and 6 mg/mL MT as the lyophilization protectant. Different lowercase letters indicate significant differences (*p* < 0.05).

The higher the extracellular β-GAL enzyme activity, the greater the cell membrane damage [14]. As shown in Figure 3, the normal strain group (0.0414 ± 0.0014 U/10^4^ cell) had the lowest extracellular β-GAL activity before freeze-drying. The β-GAL concentration in the supernatant of the reconstituted freeze-dried powder increased, indicating that the cell membranes of *Lactobacillus plantarum* FQR were damaged to varying degrees during freeze-drying, leading to the release of β-GAL outside the cell membrane. Compared with the NaCl group (0.0748 ± 0.0095 U/10^4^ cell), the addition of different concentrations of MT could reduce the extracellular β-GAL activity, but there was no significant difference (*p* > 0.05). The MT concentration had no significant difference in the β-GAL activity of the strains after freeze-drying (*p* > 0.05), showing a trend of first decreasing and then increasing with the increase in MT concentration. Among the tested concentrations, 5 mg/mL MT showed the lowest extracellular β-GAL activity (0.0573 ± 0.0049 U/10^4^ cell). Although extracellular β-GAL activity in the MT-treated groups showed a decreasing trend compared with the NaCl group, the differences were not statistically significant (*p* > 0.05). Therefore, these results suggest that MT may alleviate membrane damage to some extent, but they do not provide conclusive evidence for significant protection against β-GAL leakage during freeze-drying.

**Figure 4:**
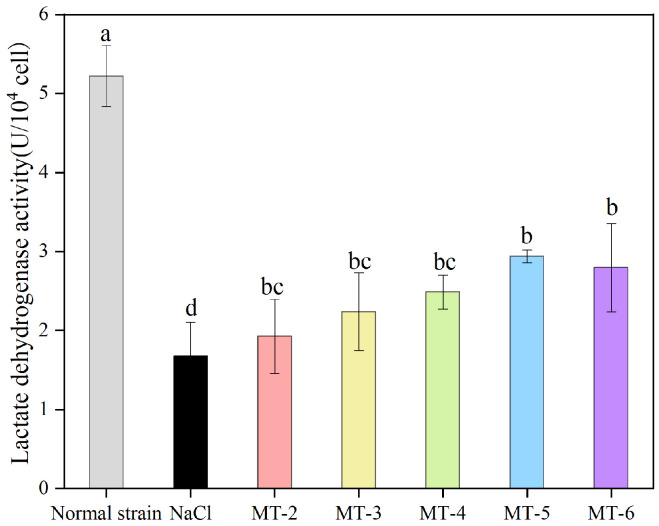
Effect of MT on intracellular lactate dehydrogenase content in *Lactobacillus plantarum* FQR cells. The normal strain represents cells without lyophilization; NaCl represents the lyophilization group with 0.9% sterile saline as the lyophilization protectant; MT-2, MT-3, MT-4, MT-5, and MT-6, respectively, represent the lyophilization groups with 2 mg/mL, 3 mg/mL, 4 mg/mL, 5 mg/mL, and 6 mg/mL MT as the lyophilization protectant. Different lowercase letters indicate significant differences (*p* < 0.05).

High LDH enzyme activity indicates that *Lactobacillus plantarum* has an intact cell membrane. As shown in Figure 4, compared with the normal strain group (5.22 ± 0.39 U/10^4^ cell), the LDH activity of *Lactobacillus plantarum* FQR after freeze-drying decreased, indicating that freeze-drying affected the LDH activity and caused damage to the strain. Compared with the NaCl group (1.68 ± 0.43 U/10^4^ cell), the addition of different concentrations of MT significantly increased the LDH activity (*p* < 0.05). The MT concentration had no significant difference in the LDH activity of the strains after freeze-drying (*p* > 0.05), showing a trend of first increasing and then decreasing with the increase in MT concentration. Among them, 5 mg/mL of MT had the best protective effect on LDH activity (2.94 ± 0.08 U/10^4^ cell). After freeze-drying treatment, the intracellular LDH enzyme content in the MT group was higher than that in the NaCl group, indicating a lower level of cell membrane damage. The experimental results indicated that the addition of MT reduced the leakage of intracellular LDH and cell membrane damage, thereby enhancing the survival ability of the strain.

**Figure 5:**
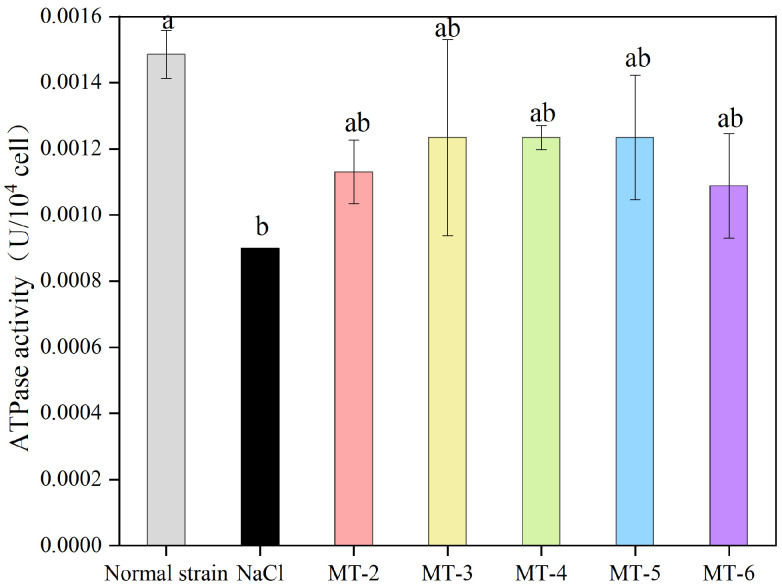
Effect of MT on intracellular Na^+^K^+^- ATPase content in *Lactobacillus plantarum* FQR cells. The normal strain represents cells without lyophilization; NaCl represents the lyophilization group with 0.9% sterile saline as the lyophilization protectant; MT-2, MT-3, MT-4, MT-5, and MT-6, respectively, represent the lyophilization groups with 2 mg/mL, 3 mg/mL, 4 mg/mL, 5 mg/mL, and 6 mg/mL MT as the lyophilization protectant. Different lowercase letters indicate significant differences (*p* < 0.05).

As shown in Figure 5, the normal strain group (0.00149 ± 0.00007 U/10^4^ cell) had the highest intracellular Na^+^K^+^-ATPase activity before freeze-drying. The Na^+^K^+^-ATPase activity of *Lactobacillus plantarum* FQR decreased after freeze-drying, indicating that freeze-drying affected the activity of Na^+^K^+^-ATPase and caused damage to the strain. Compared with the NaCl group, the addition of different concentrations of MT increased the activity of Na^+^K^+^-ATPase, but there was no significant difference (*p* > 0.05). The concentration of MT had no significant difference in the Na^+^K^+^-ATPase activity of the strain after freeze-drying (*p* > 0.05), among which 5 mg/mL of MT had the best protective effect on the activity of Na^+^K^+^-ATPase (0.00123 ± 0.00019 U/10^4^ cell). After freeze-drying treatment, the intracellular Na^+^K^+^-ATPase content in the MT group was higher than that in the NaCl group, showing a lower level of cell damage. The experimental results indicated that the addition of MT reduced the leakage of intracellular Na^+^K^+^-ATPase and cell damage, promoted the transport of ATP by increasing the transmembrane potential of the strain, maintained the dynamic balance of pH inside and outside the cell, and enhanced the survival ability of the strain.

#### 3.1.3. Effect of MT on Membrane Fluidity of *Lactobacillus plantarum* FQR

**Figure 6:**
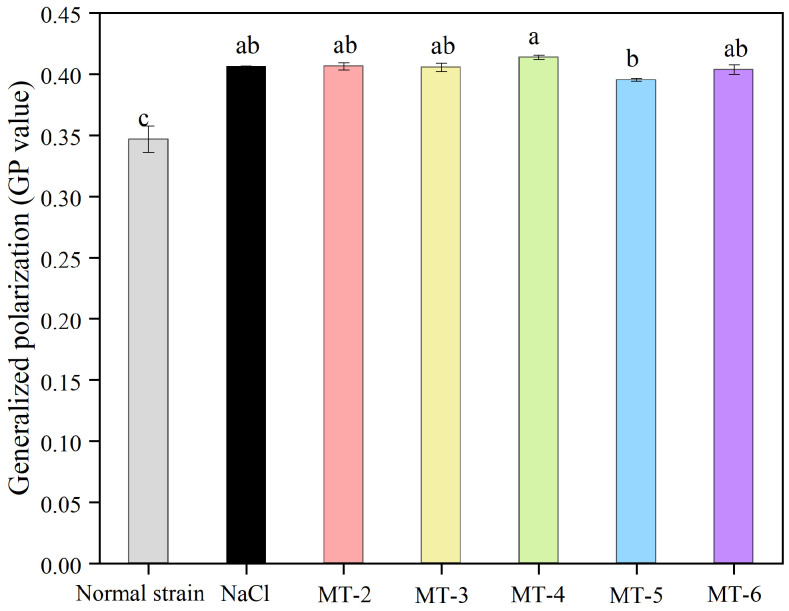
Effect of MT on membrane fluidity of *Lactobacillus plantarum* FQR cells. The normal strain represents cells without lyophilization; NaCl represents the lyophilization group with 0.9% sterile saline as the lyophilization protectant; MT-2, MT-3, MT-4, MT-5, and MT-6, respectively, represent the lyophilization groups with 2 mg/mL, 3 mg/mL, 4 mg/mL, 5 mg/mL, and 6 mg/mL MT as the lyophilization protectant. Different lowercase letters indicate significant differences (*p* < 0.05).

The fluidity of the cell membrane refers to the viscosity of its phospholipid bilayer, which is necessary for maintaining normal biological activity. This viscosity affects the movement and diffusion of biomolecules such as proteins and lipids, thereby influencing the life activities of cells [15] The fluidity of the cell membrane is negatively correlated with the GP value; the lower the GP value, the higher the fluidity of the cell membrane. As shown in Figure 6, the normal strain group (0.3468 ± 0.0109) had the lowest GP value before freeze-drying, and all freeze-dried samples showed an increase in the GP value, indicating that the freeze-drying process reduces the membrane fluidity of *Lactobacillus plantarum* FQR. After vacuum freeze-drying, there was no significant change in the GP value of the MT group compared to the NaCl group (0.4059 ± 0.0006) (*p* > 0.05). There was no significant difference in the GP value of the strains after freeze-drying with different MT concentrations (*p* > 0.05), and the GP value showed a stable trend with the increase in MT concentration, with the lowest GP value at 5 mg/mL of MT (0.3952 ± 0.0012). The above results indicate that MT did not significantly enhance membrane fluidity during freeze-drying under the tested conditions. Therefore, the improved survival rate observed in the MT group may not primarily depend on changes in membrane fluidity, but rather on the maintenance of membrane integrity, reduction in membrane permeability, and protection of intracellular physiological functions.

#### 3.1.4. Effect of MT on the Degree of Damage of *Lactobacillus plantarum* FQR

**Figure 7:**
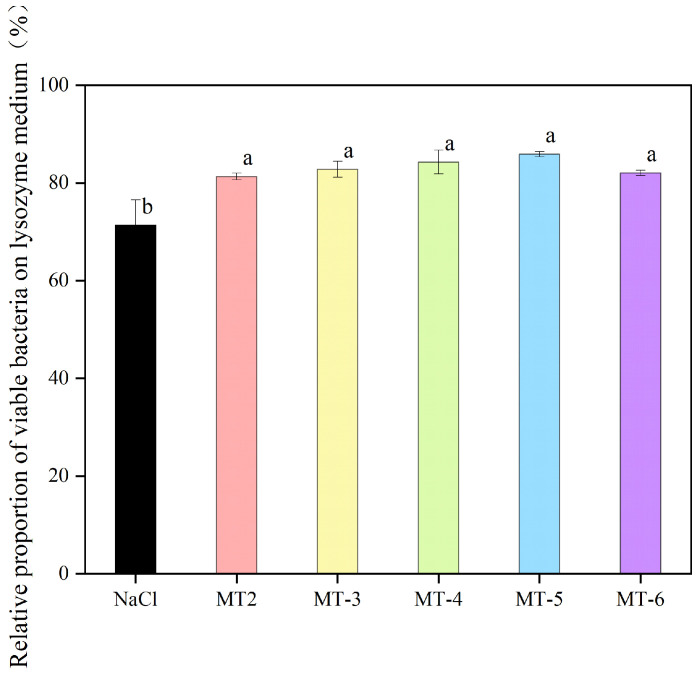
Effect of MT on the integrity of the cell wall of *Lactobacillus plantarum* FQR. NaCl represents the lyophilization group with 0.9% sterile saline as the lyophilization protectant; MT-2, MT-3, MT-4, MT-5, and MT-6, respectively, represent the lyophilization groups with 2 mg/mL, 3 mg/mL, 4 mg/mL, 5 mg/mL, and 6 mg/mL MT as the lyophilization protectant. Different lowercase letters indicate significant differences (*p* < 0.05).

As shown in Figure 7, compared with the NaCl group (71.33 ± 5.24%), the MT group exhibited a significantly higher relative proportion of viable bacteria on lysozyme-containing selective medium (*p* < 0.05). The effect of MT concentration on the cell membrane permeability of freeze-dried *Lactobacillus plantarum* FQR was not significantly different (*p* > 0.05). As the MT concentration increased, the relative proportion of viable bacteria showed a trend of first increasing and then decreasing. The relative proportion of viable bacteria was highest at 5 mg/mL MT (85.90 ± 0.53%), indicating the best cell wall protection effect. Experimental results indicate that MT addition reduces cell wall damage and better maintains cell wall integrity, thereby improving survival rates after freeze-drying.

**Figure 8:**
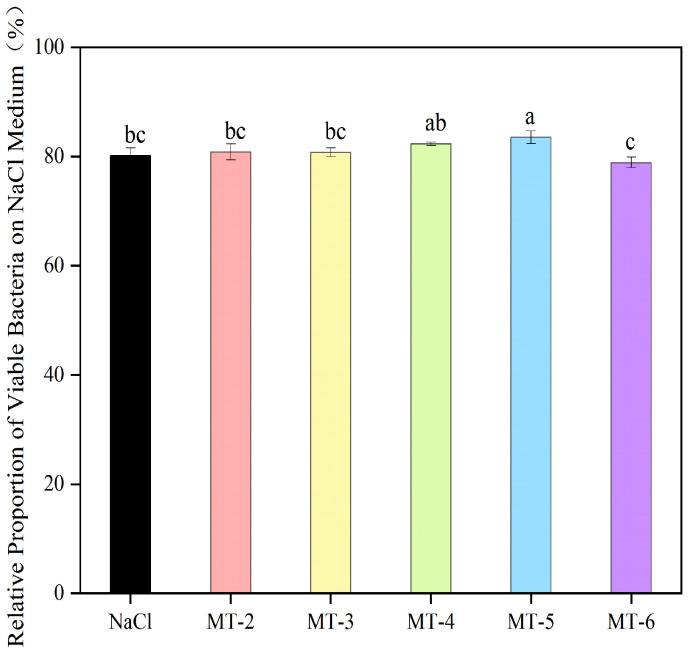
Effect of MT on membrane permeability of *Lactobacillus plantarum* FQR cells. NaCl represents the lyophilization group with 0.9% sterile saline as the lyophilization protectant; MT-2, MT-3, MT-4, MT-5, and MT-6, respectively, represent the lyophilization groups with 2 mg/mL, 3 mg/mL, 4 mg/mL, 5 mg/mL, and 6 mg/mL MT as the lyophilization protectant. Different lowercase letters indicate significant differences (*p* < 0.05).

As shown in Figure 8, compared with the NaCl group (80.16 ± 1.39%), the MT group exhibited a significantly higher relative proportion of viable cells on NaCl-limited medium (*p* < 0.05). The effect of MT concentration on the cell membrane permeability of *Lactobacillus plantarum* FQR after freeze-drying showed significant differences (*p* < 0.05). As the MT concentration increased, the relative proportion of viable bacteria first rose and then declined, with the highest relative proportion (82.35 ± 0.32%) observed at 5 mg/mL MT, indicating the best cell membrane protection effect. Experimental results indicate that MT addition effectively reduces cell membrane permeability, helps maintain membrane integrity, prevents Na^+^ and Cl^−^ influx into cells, and enhances post-freeze-drying survival rates.

**Figure 9:**
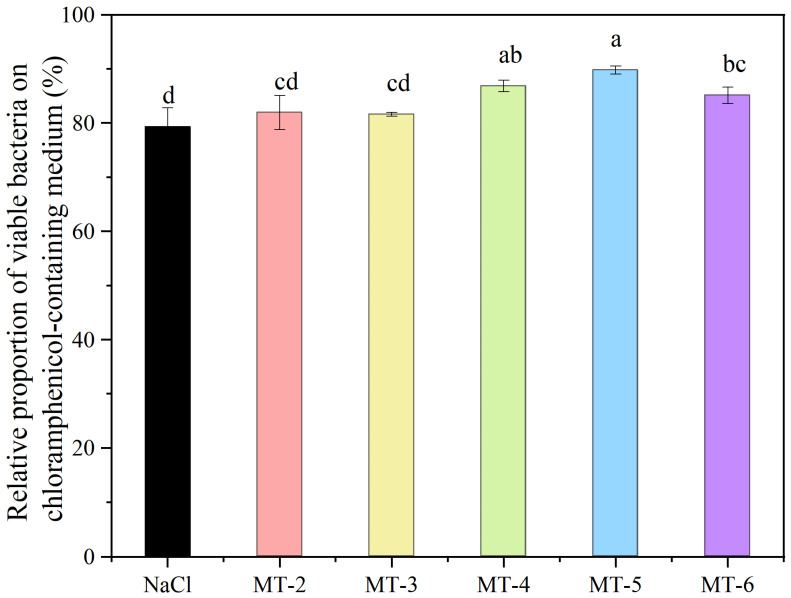
The effect of MT on the protective effect of protein synthesis in *Lactobacillus plantarum* FQR. NaCl represents the lyophilization group with 0.9% sterile saline as the lyophilization protectant; MT-2, MT-3, MT-4, MT-5, and MT-6, respectively, represent the lyophilization groups with 2 mg/mL, 3 mg/mL, 4 mg/mL, 5 mg/mL, and 6 mg/mL MT as the lyophilization protectant. Different lowercase letters indicate significant differences (*p* < 0.05).

As shown in Figure 9, compared with the NaCl group (79.34 ± 3.46%), the MT group exhibited a significantly higher relative proportion of viable bacteria (*p* < 0.05) on chloramphenicol-containing medium. The effect of MT concentration on protein synthesis in *Lactobacillus plantarum* FQR during freeze-drying exhibited significant differences (*p* < 0.05). As MT concentration increased, the relative proportion of viable cells first rose and then declined, with the highest relative proportion (89.78 ± 0.74%) observed at 5 mg/mL MT, indicating the strongest protective effect on cellular protein synthesis. Experimental results indicate that MT supplementation effectively maintains cellular protein synthesis and enhances the survival capacity of the strain after freeze-drying.

### 3.2. Research on the Antifreeze Protection Mechanism of MT on Lactobacillus plantarum FQR

#### 3.2.1. Effect of MT on Moisture CONTENT of Freeze-Dried Powder

This study analyzed the moisture content of freeze-dried *Lactobacillus plantarum* FQR supplemented with MT as a lyophilization protectant. Results shown in Table 1 indicate that MT concentration significantly influenced the moisture content of freeze-dried bacterial powder (*p* < 0.05). Specifically, as MT concentration increased, the moisture content of the freeze-dried bacterial powder first rose and then decreased. The moisture content in the MT group ranged from 7.96 ± 0.30% to 11.2 ± 0.07%, showing a statistically significant difference (*p* < 0.05) compared to the NaCl group (6.01 ± 0.36%). Typically, a moisture content of 10% in dried bacterial powders is considered a critical threshold for maintaining cell viability, while a moisture content below 5% is regarded as an ideal condition for extending product shelf life [16]. However, Üçok et al. found that storing cultures under vacuum conditions and the effectiveness of the cryoprotectant used allowed freeze-dried bacterial powder samples to exhibit high viability during storage even when their moisture content exceeded the ranges specified in the literature [17].

#### 3.2.2. Effect of MT on Moisture Distribution of Freeze-Dried Powder

**Figure 10:**
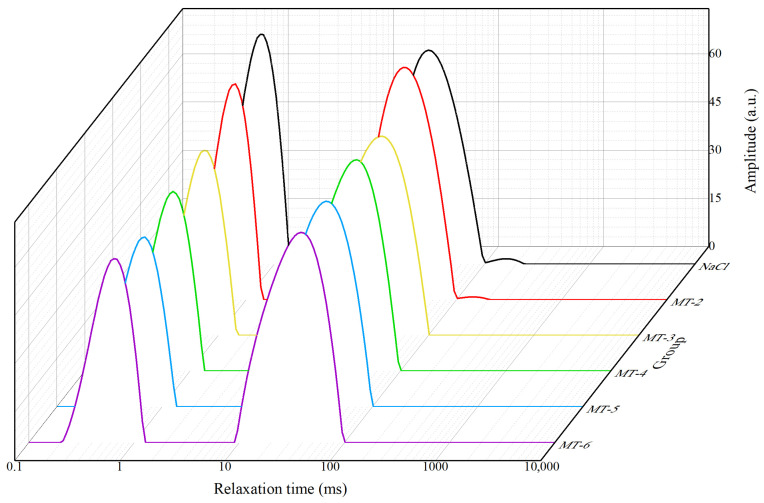
Effects of MT on the water distribution of freeze-dried bacterial powder. NaCl represents the lyophilization group with 0.9% sterile saline as the lyophilization protectant; MT-2, MT-3, MT-4, MT-5, and MT-6, respectively, represent the lyophilization groups with 2 mg/mL, 3 mg/mL, 4 mg/mL, 5 mg/mL, and 6 mg/mL MT as the lyophilization protectant.

To investigate the effects of different MT concentrations on the water state of freeze-dried bacterial powder, this study continuously measured the T2 relaxation time of samples using low-field nuclear magnetic resonance (LF-NMR) technology. Table 2 shows that the peak areas for A21 and A22 are relatively large, indicating that water in the freeze-dried bacterial powder primarily exists as bound water (T21) and non-mobile water (T22). For T21, the peak relaxation time in the NaCl group was 0.77 ± 0.03 ms, with peak area and peak ratio of 971.21 ± 76.97 and 41.57 ± 3.23%, respectively. In the MT group, the relaxation time shifted to the left, indicating reduced water mobility and decreased degrees of freedom in the sample, suggesting tighter binding and difficulty for water to leave the cells [18]. Correspondingly, the peak intensity, peak area, and peak ratio decreased in the sample, reflecting reduced bound water content. For T22, the peak relaxation time in the NaCl group was 30.79 ± 2.52 ms, with peak area and peak ratio of 1364.90 ± 73.41 and 58.43 ± 3.23%, respectively. For the MT group, the relaxation time shifted to the right, with corresponding increases in peak intensity, peak area, and peak ratio. This indicates an increase in the immobile water content of the sample and looser binding. The thermodynamic changes in freezing behavior at different MT concentrations are further demonstrated in Figure 10.

#### 3.2.3. Effects of MT on Freezing and Melting Behavior

**Figure 11:**
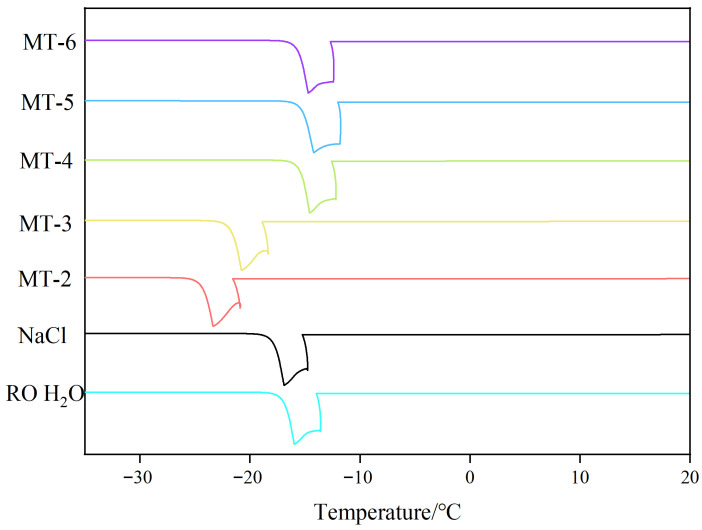
Freezing temperature of MT with different concentrations. RO H_2_O represents deionized water; NaCl represents 0.9% normal saline; MT-2, MT-3, MT-4, MT-5, and MT-6 represent MT solutions with concentrations of 2 mg/mL, 3 mg/mL, 4 mg/mL, 5 mg/mL, and 6 mg/mL, respectively. (*p* < 0.05).

As shown in Table 3, during the cooling crystallization process, the pure water (RO H_2_O) group exhibited a freezing temperature of −15.38 ± 1.01 °C and a melting temperature of 4.49 ± 0.16 °C. The NaCl group recorded a freezing temperature of −16.64 ± 0.59 °C and a melting temperature of 3.61 ± 0.63 °C. For the MT group, the minimum freezing temperature of −23.56 ± 0.67 °C was significantly lower than that of both the RO H_2_O and NaCl groups (*p* < 0.05). Significant differences existed in freezing temperature with MT concentration (*p* < 0.05), exhibiting an initial increase followed by a decrease as MT concentration rose (Figure 11). Melting temperature showed no significant difference compared to RO H_2_O or NaCl groups (*p* > 0.05). Results indicate MT inhibits ice crystal formation during solution cooling.

#### 3.2.4. Effect of MT on Ice Recrystallization

**Figure 12:**
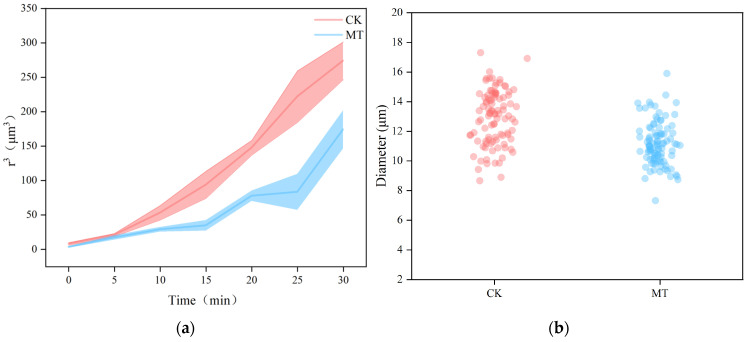
The effect of MT on ice recrystallization. (**a**) Recrystallization kinetics; (**b**) recrystallization diameter distribution, where red dots represent the CK group and blue dots represent the MT group.

As shown in Figure 12a, at the initial stage (0 min), the ice crystal size in the MT-added experimental group was smaller and similar to that in the CK group. However, after 5 min of retention, the ice crystal morphology in the MT experimental group changed from irregular to spherical or elliptical, indicating that MT significantly affects ice crystal morphology. As shown in Figure 12b, both groups exhibited increased ice crystal volume with extended annealing time. However, the MT group demonstrated a slower growth rate, reduced ice crystal size compared to the CK group, and a more concentrated size distribution [19]. The results demonstrate that MT exhibits significant ice recrystallization inhibition activity, effectively suppressing ice crystal growth, altering their morphology, and preventing small ice crystals from coalescing into large ones. Consequently, MT addition reduces damage to cellular tissues caused by large ice crystals, thereby preserving cellular viability [20]. The inhibitory effect of MT on ice recrystallization may be related to its physicochemical interactions with surrounding water molecules. Previous studies have demonstrated that MT can form hydrogen-bonding interactions with water molecules through its indole ring, amide group, and methoxy group, thereby influencing the local hydrogen-bonding network and solvation behavior of water [21,22]. These interactions may partially disrupt the ordered arrangement of water molecules during freezing, thereby inhibiting ice crystal nucleation and recrystallization. In addition, MT has been reported to strongly interact with membrane lipid interfaces, which may further contribute to membrane stabilization during freeze-drying stress [2].

#### 3.2.5. Effect of MT on the Structure of *Lactobacillus plantarum* FQR

Regarding DNA structure, prior to freeze-drying, the strain exhibited an absorption peak at 1072.83 cm^−1^ [14]. After freeze-drying, the peak position shifted to 1064.49 cm^−1^ in the NaCl group, while the MT group showed peak positions at 1068.12 cm^−1^, 1068.12 cm^−1^, 1066.67 cm^−1^, and 1068.84 cm^−1^, and 1065.58 cm^−1^. Compared to the NaCl group, the MT group largely maintained peak intensity with minimal shift in peak positions, indicating that MT at an appropriate concentration better preserves DNA structural stability [14].

In studies of cell membrane structure, the peak position for the normal strain was 1236.23 cm^−1^. After freeze-drying, the peak positions were 1235.23 cm^−1^ for the NaCl group and 1235.14 cm^−1^, 1235.14 cm^−1^, 1235.14 cm^−1^, 1235.38 cm^−1^, and 1235.22 cm^−1^. After freeze-drying, the peak positions of the NaCl and MT groups were similar, with peak intensities uniformly weakened, indicating significant changes in the cell membrane structures of both the control and experimental groups [14].

**Figure 13:**
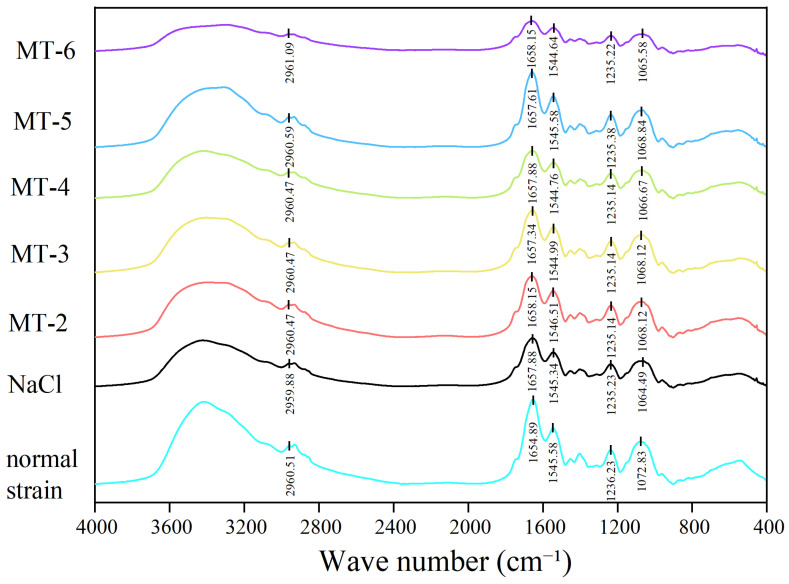
Effects of MT on the structure of *Lactobacillus plantarum* FQR cells. The normal strain represents cells without lyophilization; NaCl represents the lyophilization group with 0.9% sterile saline as the lyophilization protectant; MT-2, MT-3, MT-4, MT-5, and MT-6 respectively represent the lyophilization groups with 2 mg/mL, 3 mg/mL, 4 mg/mL, 5 mg/mL, and 6 mg/mL MT as the lyophilization protectant.

Regarding cell wall protein structure analysis, Figure 13 shows that for the amide I band: the normal strain exhibited peak positions at 1654.89 cm^−1^; the NaCl group at 1657.88 cm^−1^; and the MT group at 1658.15 cm^−1^, 1657.34 cm^−1^, 1657.88 cm^−1^ and 1657.61 cm^−1^, while the MT group showed peaks at 1658.15 cm^−1^, 1544.99 cm^−1^, 1544.76 cm^−1^, 1545.58 cm^−1^, and 1544.64 cm^−1^. For the amide II band, the normal strain exhibited a peak at 1545.58 cm^−1^, the NaCl group at 1545.34 cm^−1^, and the MT group at 1546.51 cm^−1^, 1544.99 cm^−1^, 1544.76 cm^−1^, 1545.58 cm^−1^, and 1544.64 cm^−1^. After freeze-drying, the MT group showed peaks at 1545.58 cm^−1^, 1544.76 cm^−1^, 1545.58 cm^−1^, and 1544.64 cm^−1^. 1544.99 cm^−1^; 1544.76 cm^−1^; 1545.58 cm^−1^; 1544.64 cm^−1^. After freeze-drying, the peak positions of the NaCl and MT groups became similar, with peak intensities uniformly weakened, indicating significant changes in the cell wall protein structures of both groups [14].

When examining fatty acid-related structures in the cell membrane, the results showed peak positions of 2960.51 cm^−1^ for the control group, 2959.88 cm^−1^ for the NaCl group, and 2960.47 cm^−1^, 2960.47 cm^−1^, 2960.47 cm^−1^, and 2961.09 cm^−1^ for the MT group. Although peak intensities were similar across groups, the MT group exhibited less peak shift than the NaCl group indicating that the MT group better maintained the stability of the unsaturated fatty acid structure in the cell membrane compared to the NaCl group [14].

#### 3.2.6. Metabolomics Analysis

##### Analysis of Differential Metabolites

**Figure 14:**
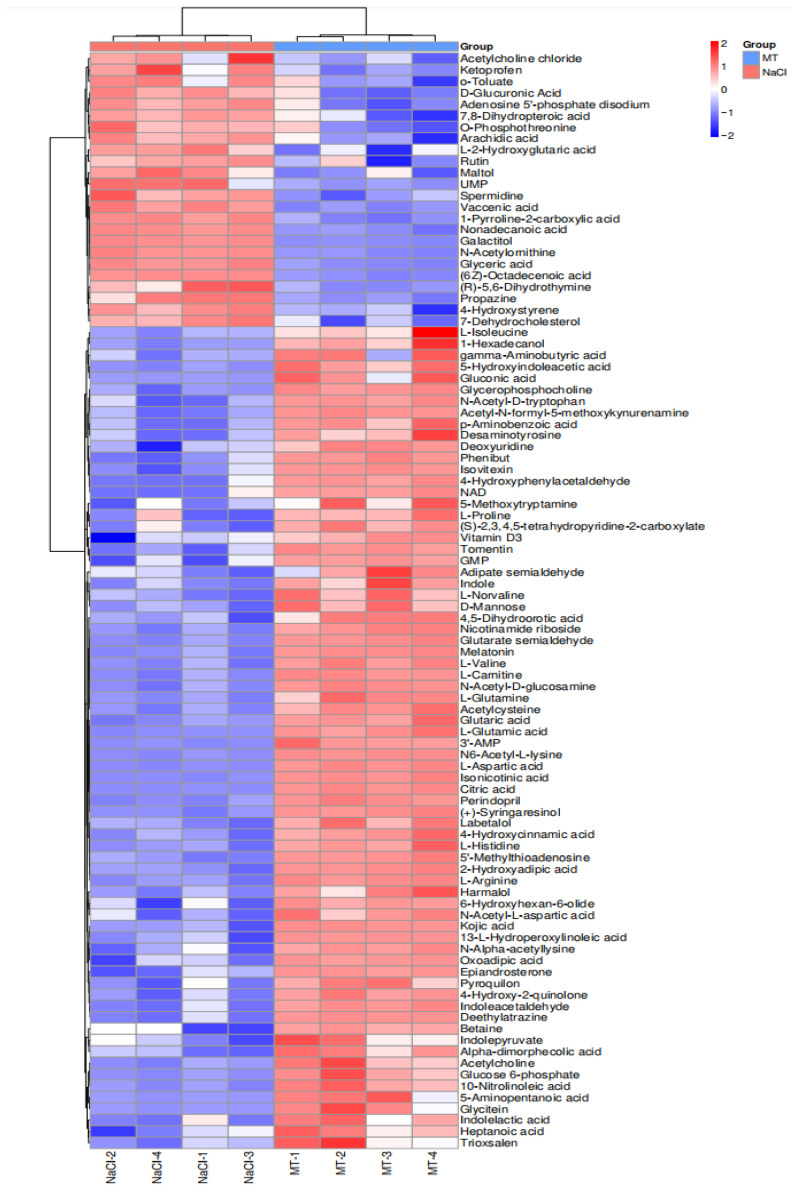
Metabolite clustering between MT group and NaCl group samples. NaCl represents the lyophilization group with 0.9% sterile saline as the lyophilization protectant; MT represents the lyophilization group with 5 mg/mL MT as the lyophilization protectant.

In this study, significant differences were screened based on predefined *p*-values and VIP scores. Metabolite profile heterogeneity among groups was examined via cluster analysis and visualized as a heatmap (Figure 14). MT-treated cells exhibited decreased levels of 72 metabolites and increased levels of 24 metabolites, with most differentially regulated metabolites associated with amino acid metabolism.

L-carnitine is a vitamin-like zwitterionic molecule associated with cold tolerance in numerous biological systems. Furthermore, L-carnitine is naturally non-toxic and found in microorganisms, plants, and most animals, serving as an essential metabolite. Zhai et al. [23] utilized L-carnitine as a cryoprotectant. Experiments demonstrated L-carnitine’s potent ability to lower water freezing points. Using an ultra-rapid freezing protocol, the thawing survival efficiency of four cell lines (GLC-82 cells, MCF-7 cells, NIH-3T3 cells, and sheep erythrocytes) was studied with L-carnitine without any organic solvent addition. At the optimal L-carnitine concentration, all four cell lines achieved survival rates exceeding 80%, significantly higher than those observed with organic cryoprotectants and trehalose. Post-cryopreservation, cellular behavior, adhesion, and proliferation resembled those of normal cells, indicating no impairment of cellular function. Furthermore, L-carnitine exhibited no apparent cytotoxicity, demonstrating superiority over organic cryoprotectants.

**Figure 15:**
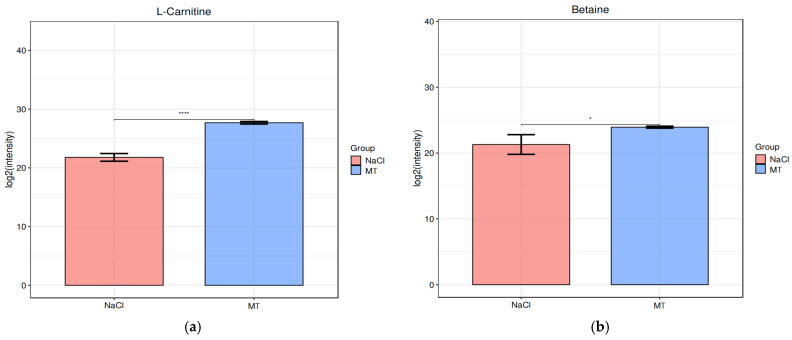
Differential metabolites between MT group and NaCl group. (**a**) Quantitative values of L-carnitine metabolites; (**b**) Quantitative values of betaine metabolites. NaCl represents the lyophilization group with 0.9% sterile saline as the lyophilization protectant; MT represents the lyophilization group with 5 mg/mL MT as the lyophilization protectant. * indicates *p* < 0.05, and **** indicates *p* < 0.0001.

Betaine, a naturally occurring zwitterionic molecule, functions as an osmoprotectant with antioxidant properties that enhance the tolerance and survival rate of lactic acid bacteria during dehydration, storage, and rehydration [24]. It is also recognized for preventing crystalline ice formation during ultra-rapid cryopreservation of HeLa, GLC-82, and MCF-10 cells, functioning as a non-toxic cryoprotectant [23]. Mori et al. [25] demonstrated that betaine effectively maintains sperm motility during freezing, improves the integrity of the sperm tail plasma membrane, and positively influences sperm motility.The quantitative differences in L-carnitine and betaine metabolites between the MT and NaCl groups are shown in Figure 15.

##### KEGG Enrichment Analysis

**Figure 16:**
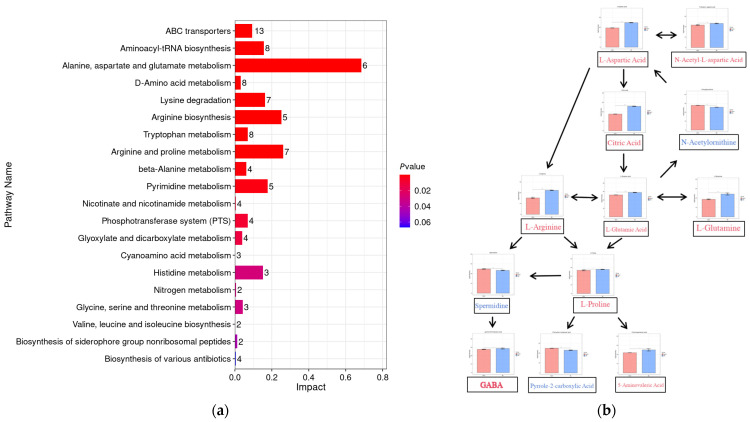
(**a**) Bar chart of metabolic pathway impact factors; (**b**) changes in intracellular amino acid metabolites (metabolites with red font are upregulated, and metabolites with blue font are downregulated). Arrows indicate the associations or conversion relationships among metabolites within the metabolic pathways.

Key metabolic pathways identified through KEGG enrichment analysis (Figure 16a) showed the highest enrichment in alanine, aspartate, and glutamate metabolism, indicating that differential metabolites in these pathways exerted the greatest influence on the target pathways. This was followed by arginine and proline metabolism. These pathways were significantly enriched (*p* < 0.05), indicating that amino acid metabolism plays an important role in MT-mediated freeze-drying protection. Based on KEGG database analysis of significantly altered metabolites, potential metabolic pathways influenced by MT addition as a lyophilization protectant were investigated. The primary metabolic shift involved amino acid metabolism. Intracellular metabolite changes in *Lactobacillus plantarum* cells were simplified into a metabolic network diagram (Figure 16b).

#### 3.2.7. Transcriptomic Analysis

As shown in Table 4, the NaCl group obtained more than 7,200,000 raw reads, and the EPS group obtained more than 6,500,000 raw reads. After screening and filtering the raw data, the Clean Reads % of both the NaCl group and the EPS group were above 98%. From the various indicators of the data, it can be seen that the sequencing quality was high, the retention rate of effective data after processing was high, and the proportion of ambiguous bases after filtering was low, all at 0.01%, meeting the requirements for subsequent analysis. All subsequent analyses were based on the Clean Reads obtained after filtering.

**Figure 17:**
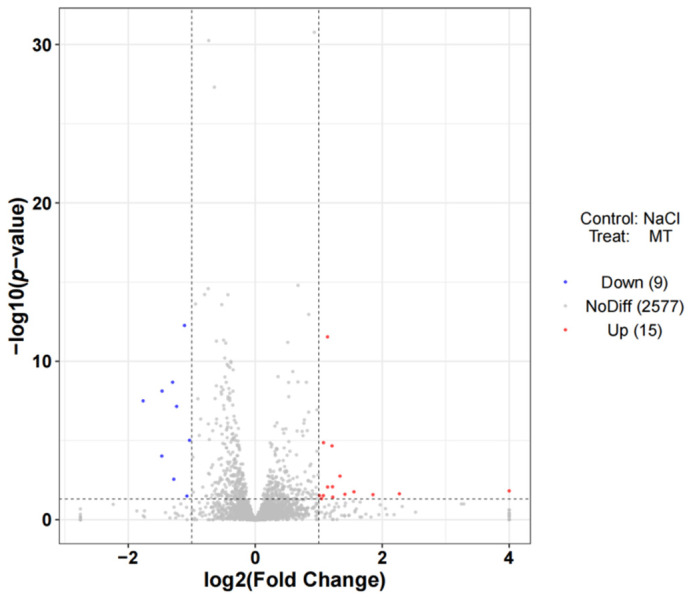
Volcano plot of differentially expressed genes between the MT and NaCl groups. The vertical dashed lines indicate the differential expression threshold (|log2FoldChange| = 1), and the horizontal dashed line indicates the statistical significance threshold (*p* = 0.05). NaCl represents the lyophilization group with 0.9% sterile saline as the lyophilization protectant; MT represents the lyophilization group with 5 mg/mL MT as the lyophilization protectant.

The distribution of differentially expressed genes is shown in Figure 17. A total of 24 genes showed significant differential expression between the MT and NaCl groups, including 15 up-regulated genes and 9 down-regulated genes, based on the criteria of |log2FoldChange| ≥ 1 and FDR < 0.05.

**Figure 18:**
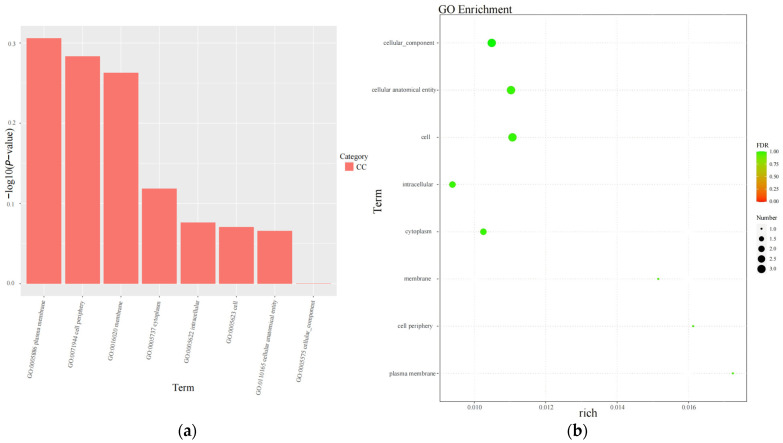
(**a**) Bar chart of enrichment analysis entries. The *x*-axis represents terms at Go level 2, and the *y*-axis shows the log10 (*p*-value) of enrichment for each term; (**b**) GO enrichment plot. The *x*-axis indicates the enrichment degree (higher Rich factor values correspond to greater enrichment), while the *y*-axis displays terms at Go level 2.

The results of GO enrichment analysis for differentially expressed genes are shown in Figure 18a. The GO classification only included the cellular component (CC). The significantly enriched GO terms (*p* < 0.05, FDR < 0.05) were mainly associated with plasma membrane organization, membrane components, and intracellular structural organization. The significantly enriched cellular components included plasma membrane (GO: 0005886), peripheral region of cell (GO: 0071944), membrane (GO: 0016020), cytoplasm (GO: 00055737), intracellular (GO: 0005622), cell (GO: 0005623), cell anatomical entity (GO: 0110165), and cell component (GO: 0005575).

Based on the GO enrichment results, the degree of enrichment was measured by the Rich factor, FDR value, and the number of genes enriched to this GO term. The Rich factor refers to the ratio of the number of differentially expressed genes enriched in this GO term to the number of differentially expressed genes annotated to this GO term. The larger the Rich factor, the greater the degree of enrichment. The FDR value generally ranges from 0 to 1, and the closer it is to 0, the more significant the enrichment. The top 20 GO terms with the smallest FDR values, indicating the most significant enrichment, were selected for display, as shown in Figure 18b.

#### 3.2.8. Non-Targeted Metabolomics and Transcriptomics Combined Analysis

**Figure 19:**
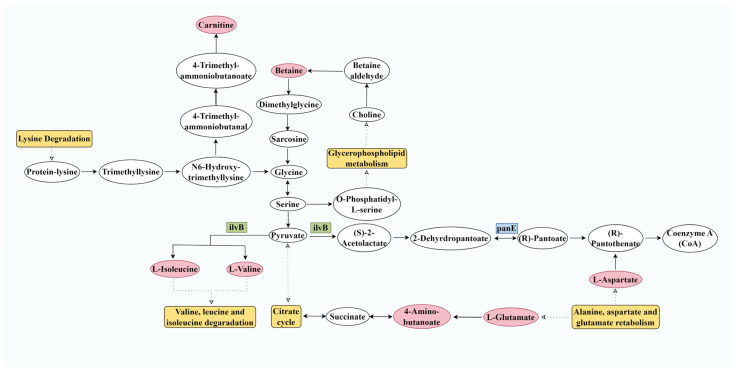
Omics joint analysis diagram. Pink ellipses represent differential metabolites, white ellipses denote non-differential metabolites involved in the pathways, yellow rectangles indicate metabolic pathway names, and green/blue boxes represent key enzyme genes that catalyze reactions. Arrows indicate metabolic relationships or reaction directions between metabolites and pathways.

Combining non-targeted metabolomics with high-throughput sequencing transcriptomics, we analyzed the dynamic changes in differentially metabolized substances and differentially expressed genes in *Lactobacillus plantarum* FQR supplemented with various lyophilization protectants. Through integrated transcriptomic and metabolomic comparisons, we identified valine, leucine, and isoleucine biosynthesis as the primary metabolic pathways. Valine, leucine, and isoleucine are branched-chain amino acids (BCAAs) that play crucial roles in lactic acid bacteria biosynthesis.The integrated omics analysis network is presented in Figure 19.

When melatonin served as a lyophilization protectant for *Lactobacillus plantarum*, its primary metabolic pathways focused on amino acid metabolism. Elevated levels of certain amino acid metabolites suggest melatonin may promote amino acid synthesis or inhibit degradation, thereby increasing intracellular accumulation of these metabolites. This increase may also indicate that cells maintain essential metabolic activities by optimizing amino acid pathways despite reduced energy demands. This optimization helps cells maintain metabolic stability during lyophilization while reducing energy expenditure, thereby effectively enhancing *Lactobacillus plantarum*’s survival rate and metabolic stability during the freeze-drying process. Specifically, the degradation capacity of lysine within metabolic pathways was optimized, leading to elevated levels of the metabolite L-carnitine. Experiments have confirmed that L-carnitine possesses potent water freezing point depression capabilities, acting as a cryoprotectant to enhance cell survival rates (Natural zwitterionic L-carnitine as an efficient cryoprotectant for solvent-free cell cryopreservation). Furthermore, melatonin supplementation optimized *Lactobacillus plantarum*’s glycerophospholipid metabolic pathway, increasing the metabolite betaine content. As a natural osmoprotectant with antioxidant properties, betaine enhances the tolerance and survival rate of lactic acid bacteria during dehydration, storage, and rehydration (Effect of protective solutes on leakage from and survival of immobilized *Lactobacillus* subjected to drying, storage and rehydration). This directly indicates that melatonin effectively protects the membrane structure and metabolic function of *Lactobacillus plantarum* by maintaining cell membrane integrity, regulating fatty acid composition, and providing metabolic support. Simultaneously, by preserving cell membrane integrity, melatonin reduces amino acid leakage, allowing intracellular accumulation of amino acid metabolites. This further corroborates the earlier experimental conclusion that melatonin mitigates freeze-drying-induced damage to cell membranes.

These transcriptomic and metabolomic results collectively suggest that MT improves freeze-drying tolerance mainly through membrane protection, amino acid metabolic regulation, and enhanced cellular stress resistance.

### 3.3. Storage Stability

Table 5 presents the relative survival rate changes in *Lactobacillus plantarum* FQR treated with different lyophilization protectants after 42 d storage at 37 °C. All groups showed a significant decline in relative survival rates post-storage, with Groups C and E demonstrating significantly higher storage stability than the NaCl group at this temperature. Notably, Group C exhibited a higher relative survival rate than Group E, indicating that melatonin supplementation enhances the strain’s relative survival rate during storage.

## 4. Discussion

### 4.1. Unique Advantages of Melatonin in LAB Cryoprotection Comparative Insights

This study establishes MT as a potent, mechanistically distinct cryoprotectant for *Lactobacillus plantarum* FQR. Its superiority is rooted not in isolated biophysical effects, but in the synergistic integration of physical stabilization, structural reinforcement, and metabolic reprogramming. Conventional agents such as trehalose and skimmed milk rely primarily on passive, extracellular mechanisms: trehalose forms vitrified hydrogen-bond networks that suppress molecular mobility, while skimmed milk provides a heterogeneous colloidal barrier via casein micelles and whey proteins. Importantly, MT is an endogenous hormone with known roles in regulating oxidative stress, membrane integrity, and intracellular signaling. In contrast, MT may exert protective effects through interactions at the membrane interface and potentially through intracellular regulatory pathways, although intracellular uptake and accumulation were not directly measured in the present study. Previous biophysical studies demonstrated that MT strongly interacts with zwitterionic lipid membranes and modulates membrane organization [2,3], supporting the membrane-protective effects observed in this study. It thereby confers comprehensive, multi-tiered protection against freeze-drying stress.

In comparison with conventional cryoprotectants such as trehalose, skim milk, and polyols, melatonin exhibits several distinct advantages. Traditional cryoprotectants mainly function through extracellular physical stabilization mechanisms, including hydrogen-bond formation, water replacement, and inhibition of ice crystal growth. In contrast, MT not only participates in physicochemical stabilization during freezing, but also exerts intracellular antioxidant and membrane-regulatory effects. Previous studies demonstrated that MT strongly interacts with lipid bilayers and stabilizes membrane structures under stress conditions [2,3]. In addition, MT has been reported to alleviate oxidative damage and improve freeze–thaw tolerance in plants, microorganisms, and animal cells by scavenging reactive oxygen species and regulating stress-response pathways [26,27,28,29]. These multifunctional properties distinguish MT from conventional cryoprotectants that primarily rely on passive physical protection mechanisms. Although the peak survival rate with MT (32.04 ± 2.14%) is numerically lower than the 87% reported for the composite protectant (10% skim milk powder + 5% sodium glutamate) in Stefanello et al. [11], this comparison must be interpreted through the lens of formulation complexity, functional scope, and translational feasibility. We further note that, despite its lower survival rate compared with some conventional cryoprotectants, MT as a standalone protectant still provides measurable cryoprotection by maintaining membrane integrity, reducing permeability, and inhibiting ice recrystallization. These properties highlight its potential as a functional component in compound formulations, offering advantages such as chemically defined composition, natural origin, GRAS-aligned safety, and ease of integration into industrial freeze-drying processes. Stefanello et al. [11]’s system requires rigorous optimization of component ratios, pH, and drying kinetics. These constraints hinder robustness across diverse LAB strains and scale-up conditions. By contrast, MT delivers consistent, dose-dependent efficacy as a single, chemically defined molecule. It enables simplified process control, reduced raw material variability, and seamless integration into existing lyophilization protocols. Critically, its natural biosynthetic origin, GRAS-aligned safety profile, and absence of residual solvents or allergenic proteins position MT as a regulatory-preferred excipient for food, nutraceutical, and pharmaceutical applications. Synthetic additives face increasing scrutiny and consumer resistance in these fields.

A defining functional advantage of MT is its demonstrated inhibition of ice recrystallization during thawing. This is a major source of irreversible membrane damage unaddressed by most conventional cryoprotectants. Time-resolved ice growth assays confirm that MT actively remodels ice crystal architecture: it suppresses dendritic branching, promotes isotropic spherical crystallization, and reduces crystal growth velocity by >40% relative to unprotected controls. This morphological control directly attenuates mechanical disruption of the cytoplasmic membrane and synergizes with the steric stabilization conferred by macromolecular protectants (e.g., EPS or whey proteins). It establishes a functionally complementary, two-pronged strategy to safeguard cellular viability throughout the entire freeze–dry–rehydrate cycle.

### 4.2. Synergistic Protection Mechanisms Bridging Structural Integrity and Metabolic Resilience

MT’s cryoprotective efficacy emerges from tightly coupled structural and metabolic interventions. These are not additive but interdependent. Structurally, MT alleviated freeze-drying-induced envelope damage, as reflected by reduced AKP/ALP leakage and increased intracellular Na^+^/K^+^-ATPase activity relative to unprotected controls. However, some membrane-related enzymatic indicators, such as extracellular β-GAL activity, did not show statistically significant differences and therefore should be interpreted as indicative trends rather than definitive evidence of membrane protection. These metrics reflect preserved membrane integrity, phospholipid stability, and functional ion-pumping capacity. These are foundational prerequisites for post-rehydration metabolic reactivation and energy homeostasis. Although MT did not significantly improve membrane fluidity in this study, the reduced membrane permeability and lower enzyme leakage suggest that MT primarily protected membrane structural integrity rather than altering membrane dynamic behavior.

Metabolically, MT drives a coordinated, adaptive rewiring of central carbon and nitrogen metabolism. These experimental observations demonstrate MT’s combined membrane and metabolic protective effects, consistent with its multifunctional role as a hormone, enhancing freeze-drying tolerance and survival of *Lactobacillus plantarum*. It induces the branched-chain amino acid (BCAA) biosynthesis pathway. This is not merely as osmolyte precursors, but as signaling molecules that activate stress-responsive kinases (e.g., BCKDH complex). Concurrently, MT elevates intracellular pools of L-carnitine (a mitochondrial membrane stabilizer and cryosolvent) and betaine (an osmoprotectant and chaperone co-factor). This results in a 3.1-fold net reduction in protein carbonylation and a 52% decrease in malondialdehyde (MDA) levels. These are direct biochemical evidence of suppressed oxidative and dehydration stress. Importantly, this metabolic shift is not compensatory but anticipatory: transcriptomic data show upregulation of sigB-regulated general stress genes prior to freezing. This indicates MT primes cells for stress rather than merely mitigating damage after onset.

Transcriptomic integration further validates this systems-level orchestration: differentially expressed genes are significantly enriched (FDR < 0.01) in Gene Ontology terms related to plasma membrane organization (GO:0005886), lipid biosynthesis (GO:0006633), and redox homeostasis (GO:0045454). Key upregulated candidates include fabZ (involved in unsaturated fatty acid synthesis), cfa (cyclopropane fatty acid synthase), and trxB (thioredoxin reductase). All are functionally linked to membrane rigidity, cold adaptation, and ROS scavenging. Thus, MT operates as a master physiological modulator: its transcriptional, metabolic, and structural effects converge to reinforce cellular integrity before, during, and after freeze-drying. It demonstrates a level of integrated resilience unmatched by conventional single-mechanism protectants.

Nevertheless, this study did not directly quantify intracellular MT accumulation or cellular uptake during freeze-drying. Therefore, the proposed intracellular regulatory effects remain inferential and require further validation through targeted uptake analysis, fluorescent labeling, or intracellular metabolite quantification in future studies.

### 4.3. Optimization of Compound Antifreeze Agent Proportion and Its Impact on Strain Performance

Vacuum freeze-drying is a commonly used method for preparing active microbial preparations. However, this process can cause significant damage to cells, making the identification of effective cryoprotectants for LAB particularly important. Typically, combining compounds with different molecular weights from two cryoprotectants can enhance protection, thereby maintaining higher activity of *Lactobacillus plantarum* during the freeze-drying process.

This study employed a composite lyophilization protectant with the following formulation: 69.80 mg/mL EPS, 4.25 mg/mL MT, and a volume-to-mass ratio of protectant to bacterial sludge of 9:10. The control groups included the single EPS treatment group (E), trehalose treatment group (Tre), skim milk treatment group (SM), and 0.9% saline treatment group (NaCl). Fermentation performance was evaluated by measuring the biomass, pH, total acid, and total sugar in the 48 h fermentation broth. Storage stability was assessed by storing the samples at 37 °C for 6 weeks, and the shelf life was predicted. The results demonstrated that the composite lyophilization protectant exhibited the fastest initial biomass growth, with rapid decreases in fermentation broth pH, increased total acid, and accelerated total sugar consumption, indicating good cell activity and favorable metabolic characteristics. In summary, this product offers advantages such as convenient use and transportation, long storage duration, high bacterial activity, stable quality, and excellent fermentation performance. It also provides a theoretical basis for developing EPS and MT from *Lactobacillus bulloides* S1 as lyophilization protectants for the preparation of LAB powder.

## 5. Conclusions

MT, as a highly effective natural cryoprotectant, protects the integrity of cell walls and membranes during freeze-drying by reducing damage to these structures. It was associated with higher retention of intracellular enzyme activities after freeze-drying and lower membrane permeability, significantly enhancing the freeze-dried survival rate of *Lactobacillus plantarum* while maintaining cellular structural stability post-freeze-drying. The addition of MT increases the moisture content of freeze-dried bacterial powders, elevates the proportion of non-free-flowing water, lowers the solution’s freezing point and inhibits ice crystal formation, possibly through physicochemical interactions with surrounding water molecules. These effects collectively enhance bacterial bioactivity and survival rates under freeze-drying conditions. Given its natural origin and generally recognized as safe (GRAS) status, MT can be safely applied in food, nutraceutical, and other biological products. With further research and development, MT-based cryoprotectants hold practical potential for industrial-scale preservation and transportation of probiotics, foods, and pharmaceuticals.

## Figures and Tables

**Table 1:** Effect of MT concentration on the moisture content of freeze-dried *Lactobacillus plantarum* FQR powder.

Group	Moisture Content (%)
NaCl	6.01 ± 0.36 ^e^
MT-2	8.17 ± 0.01 ^d^
MT-3	9.34 ± 0.10 ^b^
MT-4	11.20 ± 0.07 ^a^
MT-5	8.61 ± 0.04 ^c^
MT-6	7.96 ± 0.30 ^d^

NaCl represents the lyophilization group with 0.9% sterile saline as the lyophilization protectant; MT-2, MT-3, MT-4, MT-5, and MT-6, respectively, represent the lyophilization groups with 2 mg/mL, 3 mg/mL, 4 mg/mL, 5 mg/mL, and 6 mg/mL MT as the lyophilization protectant. Different lowercase letters indicate significant differences (*p* < 0.05).

**Table 2:** Effects of MT on the water distribution of freeze-dried bacterial powder.

				Sample		
	NaCl	MT-2	MT-3	MT-4	MT-5	MT-6
T21 (ms)	0.77 ± 0.03 ^a^	0.77 ± 0.03 ^a^	0.76 ± 0.03 ^a^	0.71 ± 0.03 ^ab^	0.66 ± 0.03 ^b^	0.72 ± 0.03 ^a^
A21	971.21 ± 76.97 ^a^	818.56 ± 81.36 ^b^	846.47 ± 21.13 ^b^	836.34 ± 24.36 ^b^	859.60 ± 10.34 ^b^	828.00 ± 3.00 ^b^
W21 (%)	41.57 ± 3.23 ^a^	35.75 ± 2.03 ^b^	37.34 ± 0.49 ^b^	36.40 ± 0.40 ^b^	37.05 ± 0.32 ^b^	36.89 ± 0.21 ^b^
T22 (ms)	30.79 ± 2.52 ^d^	32.19 ± 1.31 ^cd^	35.31 ± 1.40 ^bc^	39.65 ± 1.61 ^a^	37.85 ± 1.50 ^ab^	35.31 ± 1.40 ^bc^
A22	1364.90 ± 73.41 ^b^	1467.10 ± 19.49 ^a^	1420.23 ± 5.74 ^ab^	1460.91 ± 19.34 ^a^	1460.29 ± 19.45 ^a^	1416.32 ± 7.68 ^ab^
W22 (%)	58.43 ± 3.23 ^b^	64.25 ± 2.03 ^a^	62.66 ± 0.49 ^a^	63.60 ± 0.40 ^a^	62.95 ± 0.32 ^a^	63.11 ± 0.21 ^a^

NaCl represents the lyophilization group with 0.9% sterile saline as the lyophilization protectant; MT-2, MT-3, MT-4, MT-5, and MT-6, respectively, represent the lyophilization groups with 2 mg/mL, 3 mg/mL, 4 mg/mL, 5 mg/mL, and 6 mg/mL MT as the lyophilization protectant. Different lowercase letters indicate significant differences (*p* < 0.05).

**Table 3:** Thermodynamic properties of MT.

Group	Freezing Temperature, Solidification Pointtf/°C	Crystallization Enthalpy ΔHc/ (J/g)	Melting Temperature Tm/°C	Melting Enthalpy ΔHc/ (J/g)
RO H_2_O	−15.38 ± 1.01 ^a^	−308.65 ± 19.23 ^a^	4.49 ± 0.16 ^a^	+465.51 ± 2.93 ^a^
NaCl	−16.64 ± 0.59 ^a^	−284.80 ± 16.30 ^a^	3.61 ± 0.63 ^a^	+379.56 ± 4.92 ^a^
MT-2	−23.56 ± 0.67 ^c^	−252.85 ± 8.13 ^b^	4.13 ± 0.49 ^a^	+453.95 ± 12.91 ^c^
MT-3	−21.31 ± 1.62 ^b^	−257.54 ± 9.58 ^b^	3.76 ± 0.05 ^a^	+408.81 ± 26.23 ^b^
MT-4	−14.49 ± 0.25 ^a^	−312.39 ± 16.23 ^a^	4.23 ± 0.44 ^a^	+457.44 ± 11.47 ^a^
MT-5	−14.41 ± 1.64 ^a^	−309.29 ± 17.93 ^a^	4.11 ± 0.31 ^a^	+453.56 ± 22.46 ^a^
MT-6	−15.54 ± 0.73 ^a^	−300.35 ± 4.85 ^a^	4.29 ± 0.45 ^a^	+465.06 ± 2.63 ^a^

RO H_2_O represents deionized water; NaCl represents 0.9% normal saline; MT-2, MT-3, MT-4, MT-5, and MT-6 represent MT solutions with concentrations of 2 mg/mL, 3 mg/mL, 4 mg/mL, 5 mg/mL, and 6 mg/mL, respectively. Different lowercase letters indicate significant differences (*p* < 0.05).

**Table 4:** Quality statistics of sequencing data.

Sample	Reads No.	*n* (%)	Q20 (%)	Q30 (%)	Clean Reads (%)	Clean Data (%)
NaCl_1	7592192	0.01	99.14	97.31	99.11	99.04
NaCl_2	7402872	0.01	99.09	97.14	99.01	98.93
NaCl_3	7418460	0.01	99.11	97.20	99.06	98.98
NaCl_4	7293256	0.01	98.98	96.86	98.85	98.79
MT_1	7955700	0.01	99.13	97.25	99.05	98.96
MT_2	8267168	0.01	99.08	97.12	99.04	98.94
MT_3	6803734	0.01	99.12	97.24	99.14	99.03
MT_4	6555222	0.01	99.04	97.02	98.97	98.88

NaCl represents the lyophilization group with 0.9% sterile saline as the lyophilization protectant; MT represents the lyophilization group with 5 mg/mL MT as the lyophilization protectant. Sample: Sample name; Reads No.: Total reads; N (%): Percentage of ambiguous bases; Q20 (%): Percentage of bases with base recognition accuracy above 99%; Q30 (%): Percentage of bases with base recognition accuracy above 99.9%; Clean Reads%: Percentage of high-quality sequence reads among sequencing reads; Clean Data%: Percentage of high-quality sequence bases among sequencing bases.

**Table 5:** Relative activity of the composite fermentation agent at different times under 37 °C.

Group	Time (d)	Relative Survival Rate Cr (%)
C	0	100.00 ± 0.00 ^a^
	7	41.26 ± 0.85
	14	17.65 ± 0.27 ^d^
	21	9.18 ± 0.05 ^f^
	28	2.94 ± 0.09 ^h^
	35	1.26 ± 0.17 ^ijk^
	42	0.77 ± 0.05 ^ijk^
EPS	0	100.00 ± 0.00 ^a^
	7	26.87 ± 0.21 ^c^
	14	11.98 ± 2.22 ^e^
	21	2.94 ± 0.68 ^h^
	28	1.82 ± 0.44 ^i^
	35	0.90 ± 0.01 ^ijk^
	42	0.37 ± 0.03 ^jk^
NaCl	0	100.00 ± 0.00 ^a^
	7	1.58 ± 0.09 ^ij^
	14	0.50 ± 0.02 ^jk^
	21	0.40 ± 0.08 ^jk^
	28	0.26 ± 0.03 ^k^
	35	0.13 ± 0.01 ^k^
	42	0.05 ± 0.01 ^jk^
MT	0	100.00 ± 0.00 ^a^
	7	21.37 ± 0.42 ^bc^
	14	13.82 ± 0.28 ^cd^
	21	7.46 ± 0.23 ^d^
	28	3.18 ± 0.22 ^e^
	35	1.27 ± 0.15 ^f^
	42	0.63 ± 0.10 ^g^

C represents the lyophilized group with added compound lyophilization protectant; E represents the lyophilized group with added 69.80 mg/mL EPS as the lyophilization protectant; NaCl represents the lyophilized group with added 0.9% sterile saline as the lyophilization protectant; MT represents the lyophilized group with 5 mg/mL MT as the lyophilization protectant. Different lowercase letters indicate significant differences (*p* < 0.05).

## Data Availability

The original contributions presented in this study are included in the article. Further inquiries can be directed to the corresponding author.

## Abbreviations

The following abbreviations are used in this manuscript:

MT	Melatonin
EPS	Exopolysaccharides
LAB	Lactic acid bacteria
ROS	Reactive oxygen species
AKP/ALP	Alkaline phosphatase
β-GAL	β-Galactosidase
LDH	Lactate dehydrogenase
CK	Control check
